# P58. Can T-cells predict response to intravesical BCG immunotherapy in high-risk non-invasive bladder cancer

**DOI:** 10.1186/2051-1426-2-S2-P32

**Published:** 2014-03-12

**Authors:** S Jallad, P Thomas, M Newport, F Kern

**Affiliations:** 1 Royal Sussex County Hospital, Urology, Brighton, UK; 2 Brighton and Sussex Medical School, Urology, Brighton, UK

## Introduction

Intravesical BCG is an example of the importance of immunotherapy in cancer treatment. It has been used since the 1970s as it has a major impact in preventing or delaying bladder cancer recurrence and possibly progression. Unfortunately 20-30% of patients who receive this treatment do not respond and they are at high risk of dying from their disease. Being able to predict response to treatment would be an invaluable tool in those patients and would help in directing them to the appropriate treatment modalities. We investigated whether immunological markers in blood can predict outcome.

## Methods

Patients with high risk non-invasive bladder cancer and due to have BCG immunotherapy were included. Blood samples were obtained before and after BCG-induction treatment. In vitro stimulation of peripheral-blood mononuclear cells with PPD which were then labelled with extra and intracellular markers in order to assess the differentiation and activation status of T-cells.

## Results

13 patients had no recurrence on follow-up cystoscopy while 6 had persistent disease. Differences were seen between the two groups in mean percentage of *Interferon gamma positive* (INFγ+) CD4 in response to PPD stimulation; 1.84% (±1.58) in the recurrence-free group versus 0.54% (±0.72) in the recurrence group [*P* value 0.0252]. This was more clear in the specialised subset [CCR7-CD27-CD28+]; 2.46% (±1.96) and 0.77% (±0.80) respectively [*P* value 0.0168]. And in the [CCR7-CD27-CD28+] subset; 5.07% (±4.19) and 1.12% (±1.25) respectively [*P* Value 0.0067]. The mean percentages of pre-treatment *tumour necrosis factor positive* (TNF+) cells were also significantly different in the [CCR7-CD27-CD28+] subset; 8.82% (±6.06) and 3.10% (±3.81) respectively [*P* value 0.0246].

## Conclusion

In bladder cancer immunotherapy, the percentage of PPD inducible INFγ+ and TNF+ CD4 cells can potentially predict outcome to treatment.

